# Eubiotic effect of rifaximin is associated with decreasing abdominal pain in symptomatic uncomplicated diverticular disease: results from an observational cohort study

**DOI:** 10.1186/s12876-023-02690-x

**Published:** 2023-03-23

**Authors:** Vladimir Ivashkin, Oleg Shifrin, Roman Maslennikov, Elena Poluektova, Alexander Korolev, Anna Kudryavtseva, George Krasnov, Nona Benuni, Giovanni Barbara

**Affiliations:** 1 Department of Internal Medicine, Gastroenterology and Hepatology, Sechenov University, Moscow, Russian Federation; 2 Scientific Community for the Human Microbiome Research, Moscow, Russian Federation; 3 Laboratory of Postgenomic Research, Engelhardt Institute of Molecular Biology, Russian Academy of Sciences, Moscow, Russia; 4 Department of Medical and Surgical Sciences, University of Bologna, IRCCS Azienda Ospedaliero-Universitaria, Bologna, Italy

**Keywords:** Abdominal pain, *Akkermansia*, Diverticular disease, Eubiotic, Rifaximin

## Abstract

**Background:**

Rifaximin effectively treats symptomatic uncomplicated diverticular disease (SUDD) and has shown eubiotic potential (i.e., an increase in resident microbial elements with potential beneficial effects) in other diseases. This study investigated changes in the fecal microbiome of patients with SUDD after repeated monthly treatment with rifaximin and the association of these changes with the severity of abdominal pain.

**Methods:**

This was a single-center, prospective, observational, uncontrolled cohort study. Patients received rifaximin 400 mg twice a day for 7 days per month for 6 months. Abdominal pain (assessed on a 4-point scale from 0 [no pain] to 3 [severe pain]) and fecal microbiome (assessed using 16 S rRNA gene sequencing) were assessed at inclusion (baseline) and 3 and 6 months. The Spearman’s rank test analyzed the relationship between changes in the gut microbiome and the severity of abdominal pain. A p-value ≤ 0.05 was considered statistically significant.

**Results:**

Of the 23 patients enrolled, 12 patients completed the study and were included in the analysis. Baseline abdominal pain levels decreased significantly after 3 (p = 0.036) and 6 (p = 0.008) months of treatment with rifaximin. The abundance of *Akkermansia* in the fecal microbiome was significantly higher at 3 (p = 0.017) and 6 (p = 0.015) months versus baseline. The abundance of *Ruminococcaceae* (p = 0.034), *Veillonellaceae* (p = 0.028), and *Dialister* (p = 0.036) were significantly increased at 6 months versus baseline, whereas *Anaerostipes* (p = 0.049) was significantly decreased. The severity of abdominal pain was negatively correlated with the abundance of *Akkermansia* (r=-0.482; p = 0.003) and *Ruminococcacea*e (r=-0.371; p = 0.026) but not with *Veillonellaceae*, *Dialister*, or *Anaerostipes*. After 3 months of rifaximin, abdominal pain was significantly less in patients with *Akkermansia* in their fecal microbiome than in patients without *Akkermansia* (p = 0.022).

**Conclusion:**

The eubiotic effect of rifaximin was associated with decreased abdominal pain in patients with SUDD.

## Introduction

Diverticula are small sac-like protrusions that form in the large intestine wall and represent the most frequent anatomical alteration of the colon. The presence of diverticula in the intestine, defined as diverticulosis, may be asymptomatic or proceed as symptomatic uncomplicated or complicated diverticular disease [1]. The main symptoms of symptomatic uncomplicated diverticular disease (SUDD) are episodes of abdominal pain without evidence of inflammation of diverticula (i.e., without diverticulitis) [2].

The pathogenesis of abdominal pain in SUDD is poorly understood [1, 2]. However, studies suggest that the bacteria that inhabit the colon (gut microbiota) may play a role in its pathogenesis [3, 4]. For example, compared with patients with asymptomatic diverticulosis, patients with SUDD had a decreased abundance of *Clostridium* cluster IX, *Fusobacterium*, and *Lactobacillaceae* [5]. Compared with healthy controls, patients with SUDD had a decreased abundance of *Porphyromonadaceae* and *Bacteroides fragilis* in their fecal microbiome [6], whereas an increased abundance of *Akkermansia muciniphila* was identified in the fecal samples of SUDD patients in a separate study [7]. Similarly, the abundance of *Enterobacteriaceae* was increased in colonic mucosa biopsies of patients with SUDD compared with patients without diverticular disease [8].

Several treatment options for SUDD have been proposed, including the use of the non-absorbable antibiotic rifaximin, which decreases both the severity of SUDD symptoms and the incidence of complications of diverticular disease [9–12].

The eubiotic (i.e., improving the composition of the gut microbiota) [13] effect of rifaximin has been reported in experimental studies in rats [14, 15] and in patients with Crohn’s disease [16], cirrhosis [17], and non-constipated irritable bowel syndrome [18].

Very few studies have investigated changes in the gut microbiome after treatment of diverticular disease with rifaximin. However, two of these studies assessed only 4–7 patients with SUDD alongside patients with other intestinal diseases (i.e., ulcerative colitis, Crohn’s disease, or irritable bowel syndrome) [19, 20], while a third study treated SUDD patients with other therapeutic approaches (i.e., fiber supplementation, mesalazine, probiotic mixture VivoMixx(R)) [21]. More recently, a larger study of 43 patients with SUDD identified significant variation in the composition of the gut microbiota in stool samples taken before versus after treatment with rifaximin [22]. However, these patients received only 7 days of treatment with rifaximin. Consequently, studies that evaluate the long-term effect of rifaximin on the gut microbiota in patients with diverticular disease are lacking.

Our study aimed to investigate changes in the fecal microbiome composition in patients with SUDD after repeated monthly treatment with rifaximin and the association of these changes with the severity of abdominal pain.

## Methods

### Ethics approval and consent to participate

This single-center, prospective, observational, uncontrolled cohort study was conducted according to the Declaration of Helsinki and approved by the Independent Interdisciplinary Ethics Committee (Resolution No. 13 dated 21.07.2017). All participants gave written informed consent.

### Patients

This study enrolled consecutive patients with exacerbation of SUDD who were aged > 18 years and attended the Clinic for Internal Medicine, Gastroenterology, and Hepatology of Sechenov University. The exacerbation of SUDD was defined as the presence of abdominal pain recorded in the lower left quadrant for > 24 h in patients with diverticulosis and absence of any complications (stenosis, abscesses, fistulas) [7]. Enrolled patients also had to have received dietary fiber for at least 6 months prior to study entry to prevent constipation as a risk factor for the development of diverticulitis and other complications of diverticular disease. The exclusion criteria were as follows: contraindications to the use of rifaximin (history of drug allergy to rifaximin), the use of rifaximin during the previous 6 months, cancer, acute complications of diverticular disease (development of acute diverticulitis and/or intestinal bleeding) during the previous 6 months, planned surgery, participation in other clinical trials, pregnancy, and breastfeeding. In addition, patients were excluded from the study analysis if they refused to continue, violated the rifaximin intake regimen, required additional treatment for SUDD, or used other antibacterial drugs.

### Intervention

All patients received rifaximin (Alpha Normix®) at a dose of 400 mg twice a day for 7 days per month for 6 months.

### Outcomes

Abdominal pain and the fecal microbiome were assessed at study inclusion (baseline) and after 3 and 6 months.

Abdominal pain was assessed on a 4-point scale: 0 = no pain; 1 = mild pain (easily tolerated); 2 = moderate pain affecting daily activities; 3 = severe pain that interferes with daily activities. The maximum score for the 2 weeks before the assessment was also considered.

The fecal microbiome was analyzed using 16S rRNA gene sequencing according to the method described by Maslennikov and colleagues [23]. Briefly, stool samples were collected by each patient in a sterile disposable container on the morning of admission and immediately frozen at -80°C [24]. DNA was isolated from the stool sample, and two rounds of PCR amplification were used to prepare libraries for sequencing. In the first round, specific primers (16S-F, TCGTCGGCAGCGTCAGATGTGTATAAGAGACAGCCTACGGGNGGCWGCAG; 16S-R, GTCTCGTGGGCTCGGAGATGTGTATAAGAGACAGGACTACHVGGGTATCTAATCC) were used to amplify the v3-v4 region of the 16S ribosomal RNA gene. During the second round of PCR, specific adapters were attached to the PCR product to enable multiplex sequencing. After measuring their concentration and quality, the prepared libraries were mixed in equal proportions, and pair-end readings of 300 + 300 nucleotides were obtained on a MiSeq (Illumina) device. Reads were trimmed from the 3’-tail with Trimmomatic (Illumina) and merged into a single amplicon with the MeFiT tool [25, 26]. Amplicon sequences were then classified using the Ribosomal Database Project (RDP) classifier and RDP database [27].

### Statistical analysis

Data are reported as median [interquartile range (IQR)]. The Mann-Whitney test was used to assess differences between continuous variables. For differences between categorical variables, the Fisher’s exact test was used. Variations in the abundance of gut microbiome taxa were analyzed using the Wilcoxon test. The Spearman’s rank test was used to assess correlations between the variables computed. A p-value ≤ 0.05 was considered statistically significant. Statistical analysis was performed using STATISTICA 10 (StatSoft Inc., USA).

## Results

**Fig. 1:**
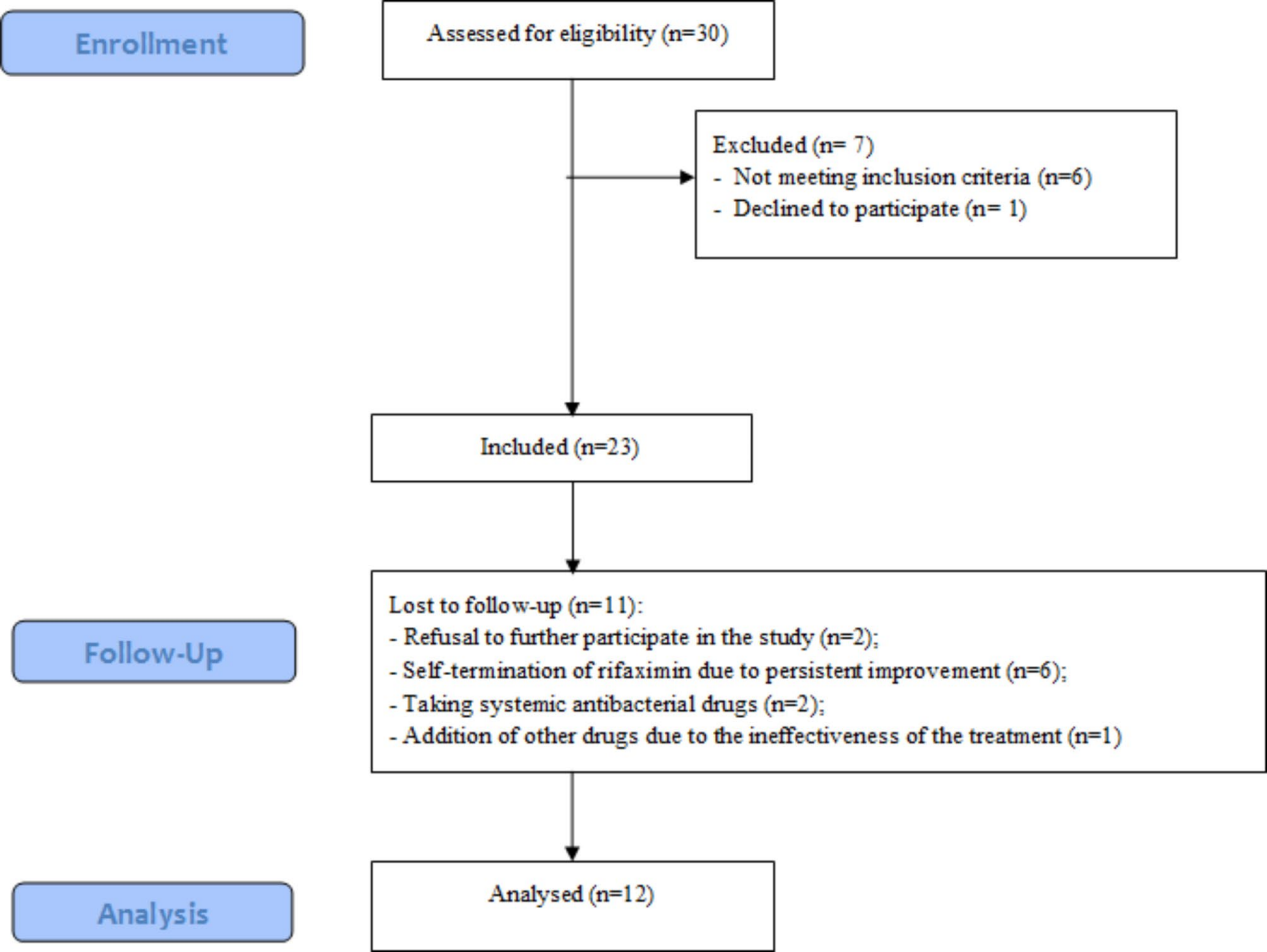
CONSORT 2010 Flow Diagram

Thirty patients were assessed for eligibility. Twenty-three patients were included in the study, and 12 patients completed the study (Fig. 1). Eleven patients were lost to follow-up; 6 (55%) patients stopped taking rifaximin due to persistent improvement, 2 patients refused to participate further in the study or took systemic antibiotics, and 1 patient required additional drugs due to the persistence of abdominal pain.

The median [IQR] age of patients who completed the study was 68 [55–71] years, body mass index was 26.4 [24.8–27.6] kg/m^2^, and 50% of patients were male. All patients were Caucasian. Complete blood counts and main biochemical blood biomarkers were normal in all patients. None of the patients had taken probiotics or antibiotics in the 6 weeks prior to study entry. Four patients received therapy for arterial hypertension. We found no evidence in the literature that these drugs have a significant effect on abdominal pain or gut microbiota. The remaining patients reported no concomitant medication.

**Fig. 2:**
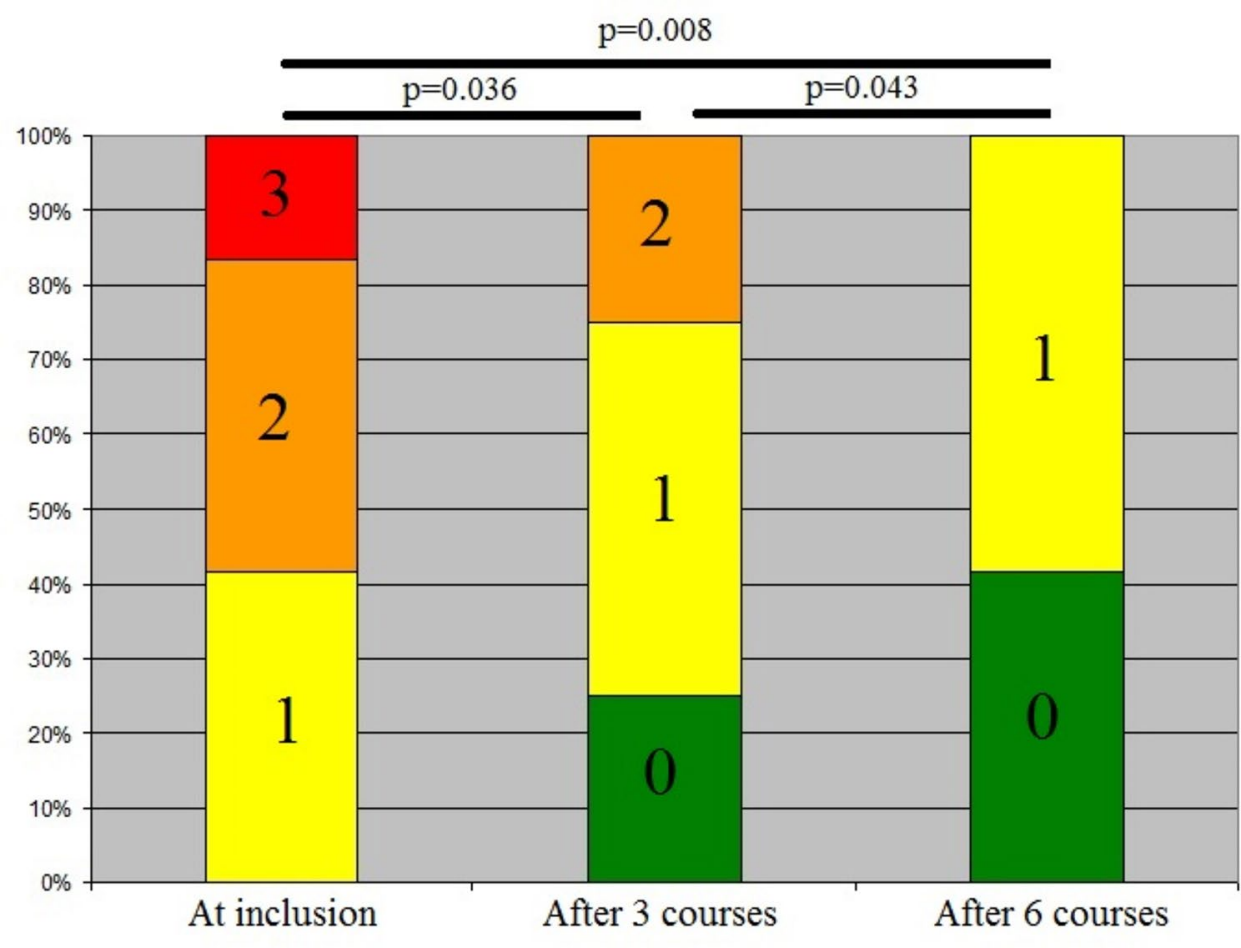
Distribution of patients according to the severity of abdominal pain (3 - severe pain; 2 – moderate pain; 1- mild pain; 0 - no pain) at inclusion, after 3 and 6 courses of rifaximin

Compared with baseline levels, significant improvement in abdominal pain was identified after 3 months of rifaximin (p = 0.036), with improvement further pronounced at 6 months (p = 0.008) (Fig. 2). None of the patients developed complications of diverticular disease or side effects from rifaximin.

Analyses of the fecal microbiome (Table 1) identified significant increases in the abundance of the phylum Verrucomicrobia and the genus *Akkermansia* (phylum Verrucomicrobia) at both 3 months (p = 0.018 and p = 0.017, respectively) and 6 months (both p = 0.015) compared with baseline levels. Significant increases were also observed at 6 months in the abundance of *Ruminococcaceae* (p = 0.034), *Veillonellaceae* (p = 0.028), and *Dialister* (p = 0.036) (family *Veillonellaceae*). In contrast, the abundance of *Anaerostipes* (p = 0.049) (family *Lachnospiraceae*) decreased significantly after 6 months of rifaximin compared with baseline levels. No significant differences in abundance were identified for the remaining taxa investigated.

**Table 1:** Changes in the fecal microbiome in patients with symptomatic uncomplicated diverticular disease after 3 and 6 months of rifaximin

Taxon	At inclusion	After 3 courses	After 6 courses	p*	p**	p***
Main phyla						
Firmicutes	68.6 [59.4–74.3]	70.4 [60.6–76.7]	72.7 [64.8–81.8]	0.814	0.638	0.814
Bacteroidetes	28.3 [23.9–38.2]	27.3 [16.2–38.1]	25.2 [15.6–33.1]	0.814	0.583	0.694
Proteobacteria	1.6 [0.8–3.9]	1.3 [0.7–2.5]	1.4 [1.2–2.4]	0.272	0.480	1.000
Actinobacteria	0.2 [0.1–0.8]	0.1 [0.0–0.7]	0.1 [0.0–1.0]	0.346	0.695	0.433
Verrucomicrobia	0.0 [0.0–0.0]	0.1 [0.0–1.0]	0.3 [0.0–0.7]	**0.018**	0.799	**0.015**
**Main classes**						
*Clostridia*	65.9 [55.1–72.5]	66.9 [55.3–74.4]	69.2 [59.8–76.7]	0.875	0.433	0.875
*Bacilli*	0.2 [0.1–0.7]	0.5 [0.1–1.6]	0.1 [0.0–1.1]	0.158	0.721	0.374
*Negativicutes*	1.2 [0.5–1.4]	1.5 [1.1–1.7]	1.5 [0.9–2.0]	0.136	0.937	0.272
**Main families**						
*Lachnospiraceae*	26.3 [18.6–32.4]	21.0 [14.1–30.3]	21.0 [13.2–30.0]	0.117	0.638	0.158
*Ruminococcaceae*	33.5 [27.3–38.0]	37.0 [30.1–41.1]	41.7 [36.5–47.4]	0.239	0.427	**0.034**
*Bacteroidaceae*	15.6 [10.8–27.4]	13.6 [6.2–29.5]	20.1 [7.6–26.8]	0.695	1.000	0.937
*Prevotellaceae*	3.81 [0.37–12.44]	2.73 [0.91–8.05]	2.66 [1.07–4.03]	0.722	0.594	0.477
*Porphyromonadaceae*	1.64 [0.82–2.66]	1.35 [0.96–1.73]	1.78 [1.33–3.44]	0.530	0.209	0.308
*Rikenellaceae*	0.97 [0.22–2.69]	0.49 [0.07–1.61]	0.61 [0.16–1.78]	0.182	0.814	0.308
*Peptostreptococcaceae*	0.63 [0.19–1.32]	0.06 [0.00–0.55]	0.19 [0.13–0.62]	0.241	0.424	0.308
*Sutterellaceae*	0.59 [0.05–0.66]	0.42 [0.21–0.64]	0.20 [0.09–0.56]	0.480	0.583	0.209
*Streptococcaceae*	0.24 [0.06–0.66]	0.23 [0.05–1.10]	0.04 [0.00–0.97]	0.480	0.721	0.514
*Veillonellaceae*	0.17 [0.00–0.45]	0.08 [0.00–1.09]	0.84 [0.00–1.83]	0.139	0.799	**0.028**
*Coriobacteriaceae*	0.09 [0.03–0.18]	0.03 [0.00–0.10]	0.09 [0.01–0.16]	0.213	0.286	0.582
*Bifidobacteriaceae*	0.08 [0.00–0.51]	0.00 [0.00–0.65]	0.03 [0.00–0.84]	0.508	0.678	0.721
*Enterobacteriaceae*	0.05 [0.00–0.72]	0.25 [0.12–0.69]	0.39 [0.00–1.46]	0.799	0.388	0.575
**Genera with a significant change in abundance**						
*Dialister*	0.00 [0.00–0.25]	0.00[0.00–1.51]	0.49 [0.00–1.46]	0.093	0.779	**0.036**
*Akkermansia*	0.00 [0.00–0.00]	0.11[0.00–1.02]	0.31 [0.01–0.70]	**0.017**	0.721	**0.015**
*Anaerostipes*	0.42 [0.18–1.06]	0.28[0.09–1.03]	0.17 [0.11–0.37]	0.929	0.272	**0.049**

Data are presented as median percentage [interquartile range]. *p-value between 3 months and baseline; **p-value between 6 months and 3 months; ***p-value between 6 months and baseline. A p-value ≤ 0.05 was considered statistically significant (highlighted in bold)

*Akkermansia* was detected in the fecal microbiome in 2 of 12 (16.7%) patients at inclusion. This increased to 7 of 12 (58.3%; p = 0.045) patients at 3 months, and 9 of 12 (75.0%; p = 0.006) patients after 6 months of rifaximin.

The patients were divided into subgroups based on the presence or absence of *Akkermansia* in the fecal microbiome at 3 months as follows; patients with *Akkermansia* (Akkermansia[+]; n = 7) versus patients without *Akkermansia* (Akkermansia[-]; n = 5).

The severity of abdominal pain was significantly less in the Akkermansia[+] group than in the Akkermansia[-] group after 3 months (median 1.0 [0.0–1.0] versus 2.0 [1.0–2.0] points, respectively; p = 0.022) and 6 months (median 0.0 [0.0–1.0] versus 1.0 [1.0–1.0] points, respectively; p = 0.023) of rifaximin, whereas no significant between-group differences were identified at baseline (median 2.0 [1.0–2.0] versus 2.0 [1.0–2.0] points, respectively; p = 0.876).

**Fig. 3:**
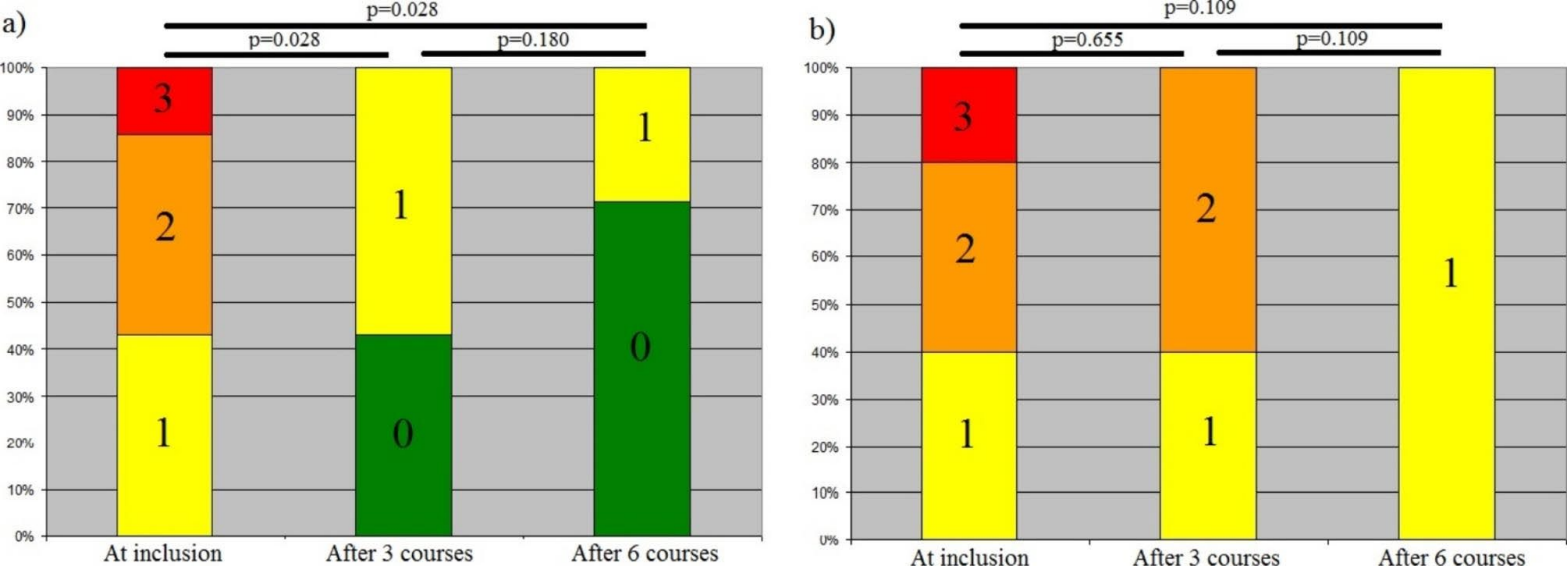
Distribution of patients according to the severity of abdominal pain (3 - severe pain; 2 – moderate pain; 1- mild pain; 0 - no pain) at inclusion, after 3 and 6 courses of rifaximin in the Akkermansia[+] (**a**) and Akkermansia[-] (**b**) groups

Significant decreases in abdominal pain were observed in the Akkermansia[+] group at 3 and 6 months compared with baseline (both p = 0.028) (Fig. 3a). The severity of abdominal pain also decreased, albeit without significance, in the Akkermansia[-] group after 6 months of rifaximin compared with 3 months (Fig. 3b), however, the decrease in abdominal pain was only observed in patients with detectable *Akkermansia* in their fecal microbiome.

The severity of abdominal pain was negatively correlated with the abundance of *Akkermansia* (r=-0.482; p = 0.003), Verrucomicrobia (r=-0.440; p = 0.007), and *Ruminococcaceae* (r=-0.371; p = 0.026) in the fecal microbiome. No significant correlation was identified between the severity of abdominal pain and the abundance of *Veillonellaceae* (p = 0.486), *Dialister* (p = 0.101), or *Anaerostipes* (p = 0.867).

## Discussion

To the best of our knowledge, this study is the first to evaluate the long-term (i.e., 6 months) effect of rifaximin on the gut microbiota in patients with SUDD. We show here that rifaximin significantly reduced the severity of abdominal pain, which is consistent with previous studies [9–12]. Whereas treatment with rifaximin over the 6 months was not accompanied by significant changes in the abundance of most major taxa of the fecal microbiome, increases in the abundance of *Akkermansia*, Verrucomicrobia, and *Ruminococcaceae* were observed and inversely correlated with the severity of abdominal pain.

Changes in the abundance of *Veillonellaceae*, *Dialister*, and *Anaerostipes* were also observed after 6 months of rifaximin, however, they were not correlated with abdominal pain severity. Thus, it is likely that these bacteria are not involved in the development of abdominal pain in SUDD.

In our study, *Akkermansia* were identified in the fecal microbiome of 2 patients (16.7%) at inclusion and in 9 patients (75.0%) after 6 months of rifaximin, whereas these bacteria were detected in 90.5% of healthy individuals in a separate study (p < 0.001) (unpublished data from [23]). However, a significantly higher abundance of *A. muciniphila* has previously been reported in fecal samples of patients with SUDD compared with healthy controls [7]. It should be noted, however, that both our study and the study by Tursi and colleagues [7] excluded patients with a recent history of acute diverticulitis. It is possible that, in patients with diverticulosis, the number of *Akkermansia* may increase as a compensatory reaction. Therefore, patients with abundant *Akkermansia* develop asymptomatic diverticulosis or SUDD, while patients who do not have high enough numbers of *Akkermansia* for this compensatory reaction develop acute diverticulitis. It should also be noted that, in the study by Tursi and colleagues [7], patients with SUDD had a lower abundance of *A. muciniphila* in the gut microbiota than patients with asymptomatic diverticulosis, however, this difference did not reach the limits of significance, which may have been due to the small patient population (15 and 13 patients, respectively). New larger studies should be performed to resolve this problem.

*Akkermansia* is the main representative of the Verrucomicrobia phylum in the gut microbiome. This bacterium has several beneficial properties, including an anti-inflammatory effect [28–32]. Specifically, the presence of *Akkermansia* increases the thickness of the mucin layer and improves the intestinal epithelial barrier, preventing the translocation of harmful bacteria and their components into the intestinal wall [31]. This bacterial translocation results in low-level inflammation, which is believed to play an important role in the pathogenesis of abdominal pain in SUDD [1]. Moreover, the intensity of infiltration of the mucous membrane of the diverticula by inflammatory cells inversely correlates with the abundance of *Akkermansia* in the mucosal microbiome [5].

The positive effect of *Akkermansia* on the epithelial barrier and mucous layer is believed to be because these bacteria degrade mucin to molecules that stimulate its formation by feedback and are used by bacteria from the family *Ruminococcaceae* that form butyrate [29, 31], which is known to strengthen the intestinal barrier [33, 34]. In our study, the abundance of *Akkermansia* and *Ruminococcaceae* increased significantly after treatment with rifaximin. However, while the increase in the abundance of *Akkermansia* was significant at both 3 and 6 months, the increased abundance of *Ruminococcaceae* was only significant after 6 months of treatment with rifaximin. This result supports the hypothesis of the synergistic effect of these two groups of bacteria on decreasing intestinal permeability, bacterial translocation, low-level inflammation, and abdominal pain associated with patients with SUDD.

Although rifaximin has been reported to increase the abundance of bacteria under the *Ruminococcaceae* family, there have been no published data to show that its use increases the abundance of *Akkermansia* [13]. In a previous study [22], rifaximin significantly altered the relative abundance of specific bacteria in patients with SUDD, with a significantly greater abundance of Bacteroidaceae, *Citrobacter*, and *Coprococcus*, and a deficiency of Mogibacteriaceae, Christensenellaceae, Dehalobacteriaceae, Pasteurellaceae, *Anaerotruncus*, *Blautia*, *Eggerthella lenta*, *Dehalobacterium*, *SMB53*, and *Haemophilus parainfluenzae* (p-adj < 0.05) reported. However, as patients received only 7 days of treatment with rifaximin, these results must be viewed with caution as they may not represent the long-term effect of rifaximin on the gut microbiota in patients with SUDD.

Two small studies investigated the difference in the gut microbiome between patients with asymptomatic diverticulosis and SUDD [5, 7]. Although neither study reported a significant between-group difference in the abundance of *Akkermansia* in the gut microbiome, counts of *A. muciniphila* species were numerically lower in patients with SUDD than in those with asymptomatic diverticulosis (-3.56 ± 1.27 versus − 3.41 ± 1.13, respectively) [7]. However, larger studies are required to confirm the hypothesis that the decreased abundance of *Akkermansia* in patients with diverticulosis is associated with their transition from asymptomatic to symptomatic. Furthermore, a cohort study of patients with asymptomatic diverticulosis and periodic analysis of their gut microbiota may identify predictors of SUDD.

All patients in our study consumed dietary fiber to prevent constipation, a risk factor for complications of diverticular disease. However, since we selected patients who had consumed dietary fiber for a minimum of 6 months before enrollment, this is unlikely to have influenced our results. In addition, we did not evaluate the severity of stool disturbances or bloating in our patients, since these may depend on dietary fiber intake.

Several limitations of the present study must be acknowledged. Firstly, the number of participants was low, and a substantial proportion of patients were lost at follow-up. Nonetheless, our preliminary results are promising and may support the design of larger controlled studies. The small number of participants can also be explained by self-termination of rifaximin due to persistent improvement or other divergences that led to the exclusion of these patients from the study analysis. Another significant limitation of our study is the lack of a placebo control arm essential to demonstrate an unambiguous symptomatic benefit of rifaximin. Large randomized controlled trials are therefore required to support our findings.

## Conclusion

In conclusion, our study is the first to describe changes in the gut microbiome after multiple courses of rifaximin to treat SUDD and the association of these changes with the severity of abdominal pain. We show significant increases in the abundance of the beneficial bacteria, *Akkermansia* and *Ruminococcaceae*, which was associated with a decrease in the severity of abdominal pain. Our study also highlights the eubiotic effect of rifaximin from as early as 3 months after starting treatment.

## Data Availability

The datasets used and/or analysed during the current study are available from the corresponding author on reasonable request.

## Abbreviations

IQR: interquartile range
SUDD: symptomatic uncomplicated diverticular disease
